# Characterization of cervical microbiota in cervical intraepithelial neoplasia and cervical cancer using low-coverage whole genome sequencing

**DOI:** 10.1128/spectrum.03206-24

**Published:** 2025-10-08

**Authors:** Tingting Zhang, Fang Gu, Weihua Li, Ruxue Han, Xinyu Liu, Chan Dai, Di Zhang, Hua Li

**Affiliations:** 1 Department of Obstetrics and Gynecology, Beijing Chaoyang Hospital Affiliated with the Capital Medical University 74639, Beijing, China; 2 Suzhou Hongyuan Biotech Inc., Biobay 676917, Suzhou, China; Pennsylvania State College of Medicine, Hershey, Pennsylvania, USA

**Keywords:** cervical cancer (CC), cervical intraepithelial neoplasia (CIN), cervical microbiota (CM), low-coverage whole-genome sequencing (LC-WGS), ultrasensitive chromosomal aneuploidy detector (UCAD)

## Abstract

This study characterized compositional shifts in cervical microbiota across disease stages from benign conditions through cervical intraepithelial neoplasia (CIN) to cervical cancer (CC) and investigated interactions with high-risk HPV (hr-HPV) infection using species-resolution profiling to identify severity-associated biomarkers. Cervical exfoliated epithelial cells from 50 patients (eight normal/CIN1, 15 CIN2, 19 CIN3, 5 CC) were analyzed using Low-Coverage Whole Genome Sequencing combined with the Ultrasensitive Chromosomal Aneuploidy Detector (UCAD), a technology featuring a two-step normalization framework that systematically converts raw microbial reads into statistically validated abundance deviations. This enables quantitative identification of pathologically relevant microbiota through cohort-wide Z-score benchmarking. Microbial diversity, differential biomarkers, and HPV-microbiota interactions were assessed using Kruskal-Wallis tests, LEfSe, and Random Forest modeling. Results revealed progressive *Lactobacillus* depletion (e.g., *Lactobacillus crispatus*: 32.9% in ≤CIN2 vs. 8.8% in CC) and enrichment of pathobionts like *Gardnerella* and *Bacteroides* with lesion severity. CC exhibited the highest microbial diversity (Shannon index: CC vs. CIN2, *P*=0.045), dominated by HPV16 (11.8%), *Bacteroides* (55.4%), and *Porphyromonas* (25.2%). LEfSe identified HPV16, HPV35, *Parvimonas micra*, and *Anaerococcus lactolyticus* as CC-specific markers, while Random Forest highlighted *Mobiluncus curtisii* (importance score=2.0) and HPV16 as key discriminators. CC microbiota showed significant Bacteroidetes enrichment (82% at class level) and reduced Firmicutes abundance. These findings suggest carcinogenesis-associated microbial restructuring, marked by *Lactobacillus* loss, anaerobic proliferation, and HPV16/35 dominance, potentially modulating disease progression. The identified signatures may inform diagnostic development and microbiome-targeted therapies.

**IMPORTANCE:**

Our study pioneers an LC-WGS/UCAD approach to characterize microbial across the spectrum from benign lesions through precancerous cervical intraepithelial neoplasia to invasive cervical carcinoma. By identifying lesion-specific microbial biomarkers and HPV-associated cofactors, this work advances mechanistic understanding of microbiota-driven oncogenesis and informs future strategies for microbiota-targeted cervical cancer prevention.

## INTRODUCTION

Cervical cancer (CC) remains a significant global health burden, with GLOBOCAN 2020 estimates indicating approximately 604,000 new CC cases and 342,000 annual deaths worldwide (1). While most human papillomavirus (HPV) infections are spontaneously cleared by host immunity, persistent high-risk HPV (hr-HPV) infection has been unequivocally established as the primary etiology of cervical intraepithelial neoplasia (CIN) and subsequent carcinogenesis (2). Emerging evidence highlights the critical role of the cervical microbiome (CM), a diverse microbial community that comprises bacteria, viruses, and fungi colonizing the cervical epithelium, in modulating HPV pathogenesis (3). Notably, compositional alterations in the CM may influence host susceptibility to HPV acquisition, persistence, and neoplastic progression (3).

In healthy cervicovaginal ecosystems, *Lactobacillus* species dominate the microbiota through lactic acid production, maintaining an optimal low pH microenvironment that inhibits pathogen overgrowth while reinforcing mucosal immunity (4, 5). Pathological dysbiosis characterized by *Lactobacillus* depletion and anaerobic bacterial proliferation (e.g., *Gardnerella, Prevotella*) initiates a cascade of detrimental events: upregulated extracellular matrix metalloproteinase inducers enhance matrix metalloproteinase-8 (MMP-8) activity, compromising epithelial barrier integrity (6). This loss of structural homeostasis facilitates HPV virion penetration into basal keratinocytes, enabling viral attachment, replication, and subsequent intraepithelial spread, which serves as a critical precursor to CIN development (6). These findings underscore the imperative to delineate CM compositional shifts across cervical lesion stages, which may unveil novel biomarkers for risk stratification and therapeutic intervention.

Methodological innovations are essential to address current limitations in CM characterization. The adoption of next-generation sequencing technology, alongside its diverse methodological approaches, has enabled culture-independent detection of a broad spectrum of microorganisms in complex samples (7). Notably, while conventional 16S rRNA sequencing is limited to bacterial genus-level identification (8), our study pioneers the integration of low-coverage whole-genome sequencing (LC-WGS) with the Ultrasensitive Chromosomal Aneuploidy Detector (UCAD) for species-level microbial profiling. By strategically adapting UCAD’s standardized pipeline, originally designed for chromosomal copy number variation analysis (9), we repurposed its percentage normalization and Z-score transformation algorithms to achieve cross-domain microbial characterization (bacteria, fungi, parasites, and viruses) at taxonomic resolution. To our knowledge, this represents the first implementation of LC-WGS/UCAD as a comprehensive diagnostic framework, enabled by customized bioinformatic innovations.

This investigation enrolled 50 patients to collect cervical exfoliated cells, with the primary objectives of systematically mapping compositional disparities in the CM across lesion progression stages, identifying keystone microbial taxa associated with dysbiosis-driven neoplastic transformation, and establishing a novel LC-WGS/UCAD-integrated analytical framework. This approach aimed to characterize lesion-specific CM signatures, provide preliminary insights into microbial dynamics during cervical carcinogenesis, and facilitate targeted biomarker discovery.

## MATERIALS AND METHODS

### Study participants and design

**Fig 1:**
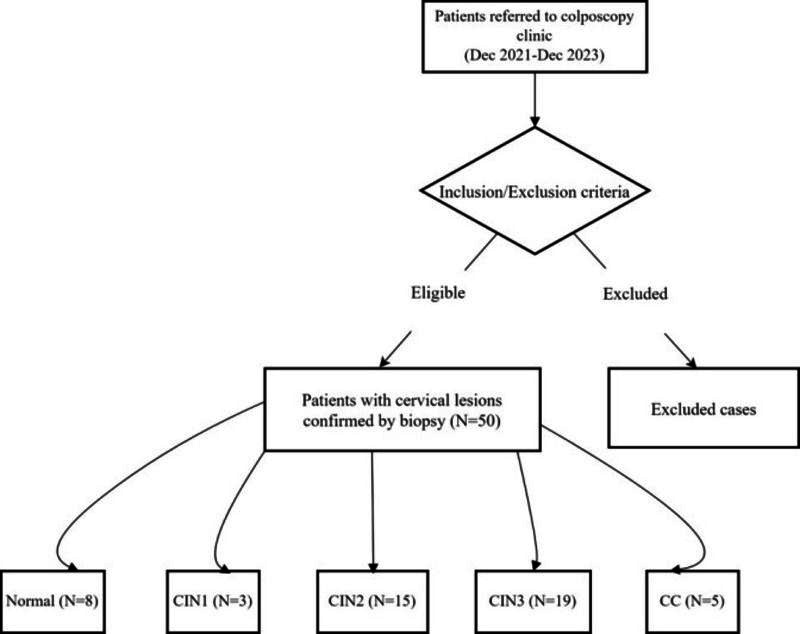
Study Flowchart.

A cross-sectional clinical study was conducted between December 2021 and December 2023 at the Colposcopy Clinic of Beijing Chaoyang Hospital affiliated with the Capital Medical University**,** enrolling 50 patients with suspected cervical lesions requiring clinical evaluation (Fig. 1). The study protocol was approved by the Institutional Review Board (IRB) of Beijing Chaoyang Hospital, and written informed consent was obtained from all participants. Demographic and clinical data, including age, family history of cancer, and gynecological history, were systematically extracted from standardized medical records.

Inclusion Criteria

Participants met the following criteria:

- Clinically suspected cervical lesions warranting colposcopy and histopathological biopsy;
- Willingness to comply with cervical specimen collection procedures.

Exclusion Criteria

Participants were excluded if they had:

- Active menstruation or pregnancy
- Prior history of cervical lesion treatment (e.g., loop electrosurgical excision procedure [LEEP], cold knife conization)
- Current genital tract infections (e.g., bacterial vaginosis, candidal vaginitis)
- Confirmed sexually transmitted infections (e.g., gonorrhea, syphilis, trichomoniasis)
- Recent antibiotic/antimicrobial use within the past 1 month, sexual intercourse, or vaginal douching within 3 days prior to sampling
- History of HPV vaccination
- Diagnosed autoimmune disorders or malignancies (excluding cervical neoplasia).

Cervical exfoliated cell specimens were collected using a cytobrush before biopsy for simultaneous HPV genotyping, ThinPrep Cytologic Test (TCT), and microbial profiling. hr-HPV testing was conducted using cervical exfoliated cell samples with the Cobas 4,800 HPV Test (Roche Diagnostics), which identifies 14 hr-HPV genotypes (16, 18, 31, 33, 35, 39, 45, 51, 52, 56, 58, 59, 66, and 68), with types 16/18 designated as very high-risk HPV (vhr-HPV) (10). Cytological interpretations were performed in accordance with the Bethesda System guidelines, with cytological findings exclusively utilized for initial risk stratification (11). Histopathological diagnoses, including normal tissue, CIN I-III, and invasive carcinoma, were established based on colposcopy-directed biopsies. These diagnoses were independently reviewed by two experienced pathologists who were blinded to the clinical data. Based on clinical behavior, CIN I, which is associated with low-risk HPV and a spontaneous regression rate of approximately 60% within 1 year, was grouped with normal findings in subsequent analyzes (12).

Total genomic DNA was extracted from cervical exfoliated cells using the Amp Genomic DNA Kit (TIANGEN). The DNA was fragmented using a Covaris ultrasonicator to generate fragments with an average size of 190 bp, and 100 ng of fragmented DNA was used to construct sequencing libraries with the NEBnext Ultra II kit. An 8 bp coding sequence adapter was ligated to the DNA fragments, followed by PCR amplification. Libraries were prepared with 12 cycles of PCR amplification, and LC-WGS with a sequencing depth of 1.0× coverage was performed via paired-end sequencing (150 bp read length) on the Illumina HiSeq X Ten platform. Each sample generated approximately 5 Gb of raw sequencing data, which underwent quality control and alignment to the human reference genome (hg19).

### Sequence data filtering

A stringent quality control pipeline was implemented to generate high-confidence clean reads:

Adapter trimming: Reads containing adapter sequences were removed using default parameters in Trimmomatic; Ambiguous base filtering: Paired-end reads were discarded if either read contained more than 10% ambiguous bases (N); Quality score filtering: Read pairs were excluded where more than 50% of bases in either read exhibited a Phred quality score <5. Filtered reads were retained for downstream analyzes only if both ends satisfied all quality thresholds, ensuring high-quality input for subsequent microbial profiling.

### Cervical microbiome bioinformatics analysis

Filtered sequencing data were mapped to the human reference genome (http://hgdownload.soe.ucsc.edu/goldenPath/hg19/bigZips/) using BWA (http://bio-bwa.sourceforge.net/bwa.shtml). Sequencing reads unmapped to the human genome were subsequently compared against reference sequences in the Human Microbiome Reference Gene Database (HMRGD), comprising bacteria, fungi, viruses, protozoa, and other pathogenic microorganisms. Based on microbial alignment results, reads corresponding to identified microbial taxa were quantified.

To balance microbial abundance (including pathogens and commensals), UCAD employed a two-step normalization pipeline: Relative abundance calculation: Raw reads for each microbial taxon were normalized against the total microbiome reads per sample to derive percentage abundances.

Z-score standardization: Relative abundances across samples were standardized using the Z-score algorithm. A Z-score ≥3 indicated significant enrichment or depletion of a microbial group relative to the cohort-wide average, reflecting substantial deviation from baseline conditions.


(1) $$Z_{microbiome}\left(sample\right)=\frac{P_{microbiome}\left(sample\right)-mean\left(P_{microbiome}\right)}{stdev\left(P_{microbiome}\right)},$$

(2) $$mean\left(P_{microbiome}\right)=\frac{\sum_{sample}P_{microbiome}\left(sample\right)}{Number\_of\_samples}\;,$$

(3) $$stdev\left(P_{microbiome}\right)=\sqrt[{2}]{\frac{\sum_{sample}{\left(P_{microbiome}\left(sample\right)-mean\left(P_{microbiome}\right)\right)}^{2}}{Number\_of\_samples-1}}.$$

### Statistical analysis

General Statistical Methods : Categorical variables were compared using Pearson’s chi-square test or Fisher’s exact test, while non-normally distributed continuous data were analyzed via the Mann-Whitney U-test (two groups) or Kruskal-Wallis test (multi-group comparisons). Pearson’s correlation coefficient was used to assess pairwise correlations between continuous variables. All analyzes were performed using SPSS v26.0 (IBM Corp.) and R v4.4.0.

Microbiome-Specific Analyzes: Alpha diversity was evaluated at the species level using the Simpson, Shannon, and Chao1 indices. Beta diversity was assessed via principal coordinate analysis (PCoA) based on Bray–Curtis dissimilarity metrics, supplemented by non-metric multidimensional scaling (NMDS) to visualize inter-group compositional differences. Multi-group differential abundance analyzes were conducted using the Kruskal-Wallis test with Dunn’s post hoc pairwise comparisons (*P*<0.05). The Linear Discriminant Analysis (LDA) Effect Size (LEfSe) algorithm was employed to identify taxa with significant differential abundance (logarithmic LDA score threshold >2.0). Random forest analysis was performed on the same data set to identify and validate key microbial biomarkers. Hierarchical clustering was used to group samples by taxonomic similarity, and stacked bar plots were generated to visualize genus-level relative abundances. Microbiome analyzes utilized the vegan and phyloseq packages in R. All statistical significance was defined as two-tailed *P* < 0.05.

## RESULTS

### Subject characteristics

This study enrolled 50 patients who visited the colposcopy clinic and met the inclusion criteria. Of these, 8 cases received benign cervicitis diagnoses, three were diagnosed with CIN I, 15 with CIN II, 19 with CIN III, and five with CC. Notably, all 5 CC cases were confirmed as squamous cell carcinomas. Based on the International Federation of Gynecology and Obstetrics 2018 staging system, the 5 CC cases were categorized as follows: stage IB2 (n=1), IB3 (n=1), IIB (n=1), and IIIC1r (n=2). Table 1 presents a summary of the cohort’s characteristics. The patients across different pathology groups exhibited comparable age distributions (P=0.31), TCT results (P=0.14), and HPV infection statuses (P=0.50). Conversely, statistically significant differences were noted in both family history and gynecological history (P<0.05).

**TABLE 1:** Baseline characteristics of the 50 Patients

Parameters	Total, n (%)	Pathology diagnosis				*P*-value
		Benign+ CIN1	CIN2	CIN3	CC	
Age						
<40y	30(60.0%)	8(26.7%)	9(30.0%)	12(40.0%)	1(3.3%)	P = 0.309
40y–59y	17(34.0%)	3(17.6%)	6(35.3%)	5(29.4%)	3(17.6%)	
>59y	3(6.0%)	0(0.0%)	0(0.0%)	2(66.7%)	1(33.3%)	
TCT results						P = 0.142
NILM	20(40.0%)	3(15.0%)	6(30.0%)	10(50.0%)	1(5.0%)	
ASC-US	12(24.0%)	5(41.7%)	4(33.3%)	3(25.0%)	0(0.0%)	
ASC-H	2(4.0%)	0(0.0%)	0(0.0%)	1(50.0%)	1(50.0%)	
LSIL	11(20.0%)	2(18.2%)	5(45.5%)	3(27.3%)	1(9.1%)	
HSIL	5(10.0%)	1(20.0%)	0(0.0%)	2(40.0%)	2(40.0%)	
HPV infection						P = 0.500
vhr-HPV(+)	27(54.0%)	6(22.2%)	5(18.5%)	12(44.4%)	4(14.8%)	
hr-HPV(+)	21(42.0%)	5(23.8%)	9(42.9%)	6(28.6%)	1(4.8%)	
hr-HPV(–)	2(4.0%)	0(0.0%)	1(50.0%)	1(50.0%)	0(0.0%)	
Family history						**P < 0.001^*a*^**
Yes	8(16.0%)	0(0.0%)	2(25.0%)	2(25.0%)	4(50.0%)	
No	42(84.0%)	11(26.2%)	13(30.9%)	17(40.5%)	1(2.4%)	
Gyn-history						**P = 0.031^*a*^**
Yes	18(36.0%)	3(16.7%)	10(55.6%)	4(22.2%)	1(5.5%)	
No	32(64.0%)	8(25.0%)	5(15.6%)	15(46.9%)	4(12.5%)	

^*a*^ The bold values indicates *P* < 0.05.

Family History includes genetic predispositions, inherited disorders, familial chronic illnesses, and other significant health-related events. Gyn history comprises gynecological benign conditions such as menstrual irregularities, endometrial hyperplasia, and endometrial polyps.

### Distribution of microorganisms in samples

**Fig 2:**
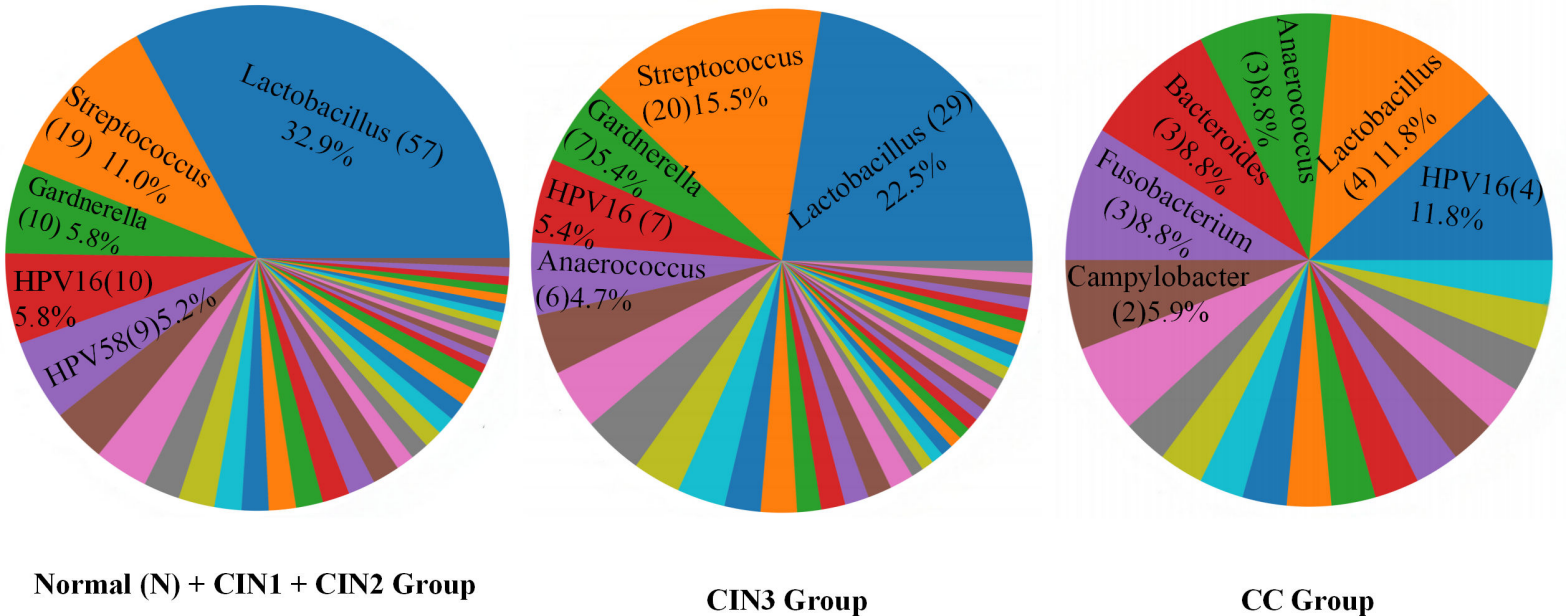
Distribution of Microorganisms in Samples from the Normal (N) + CIN1+CIN2 Combined Group, CIN3 Group, and CC Group.

Fig. 2 illustrates the declining proportion of *Lactobacillus* species as cervical lesions progress. In cases with CIN2 or less severe lesions, the predominant microorganism is *Lactobacillus* (32.9%), followed by *Streptococcus* (11.0%) and *Gardnerella* (5.8%). Among CIN3 cases, *Lactobacillus* remains the most prevalent (22.5%), followed by *Streptococcus* (15.5%) and HPV16 (5.4%). By contrast, in CC cases, HPV16 (11.8%) becomes the most abundant microorganism, with *Lactobacillus* (8.8%) and *Anaerococcus* (8.8%) following closely.

### Composition and relative abundance of the cervical microbiota across cervical lesion groups

**Fig 3:**
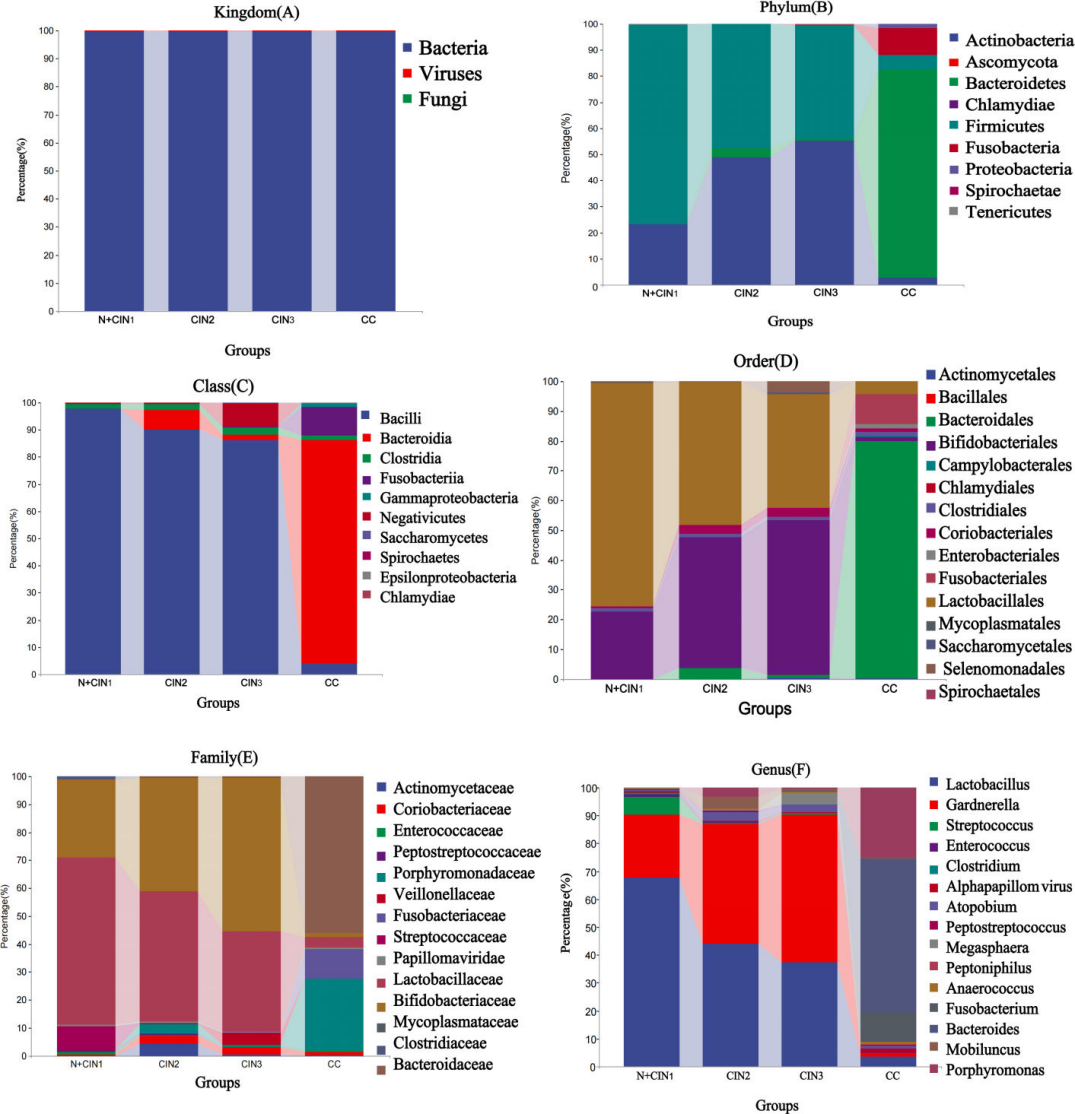
Relative Abundances of the Cervical Microbiota at the Kingdom (**A**), Phylum (**B**), Class (**C**), Order (**D**), Family (**E**), and Genus (**F**) Levels Across the N + CIN1 Group, CIN2 Group, CIN3 Group, and CC Group.

At the Kingdom level, the predominant microbial components in the cervical microbiota across all groups were Bacteria (Fig. 3A). At the phylum level, Firmicutes and Actinobacteria were the dominant phyla in the four groups (N + CIN1 combined group, CIN2 group, CIN3 group, and CC group). As cervical lesion severity increased, the relative abundance of Firmicutes decreased, while Actinobacteria showed a significant upward trend from the N + CIN1 group to the CIN3 group. Notably, the CC group exhibited markedly higher abundances of Bacteroidetes and Ascomycota compared to other groups (Fig. 3B).

At the class level, the core microbial classes in the cervical microbiota were Bacilli, Bacteroidia, and Clostridia. With lesion progression, Bacilli gradually declined, whereas Bacteroidia and Fusobacteriia increased significantly, with Bacteroidia accounting for 82% of the CC group’s microbiota (Fig. 3C).

At the order level, Lactobacillales, Bifidobacteriales, and Bacteroidales constituted the core microbial orders across all groups. The relative abundance of Lactobacillales decreased progressively with lesion severity, reaching the lowest levels in the CC group. Meanwhile, Bacteroidales showed the highest abundance in the CC group (79.7%) (Fig. 3D).

At the family level, Lactobacillaceae and Bifidobacteriaceae were the dominant families in all groups. With the progression of cervical lesions, Lactobacillaceae abundance decreased, while Bifidobacteriaceae increased from the N + CIN1 group to the CIN3 group (Fig. 3E).

At the genus level, *Lactobacillus* and *Gardnerella* were the most prevalent genera, with *Lactobacillus* showing a declining trend and *Gardnerella* an upward trend as lesions advanced. In the CC group, Bacteroides (55.44%), Porphyromonas (25.2%), and Fusobacterium were the predominant genera, with all three exhibiting lower abundances in the other three groups (N + CIN1, CIN2, and CIN3) (Fig. 3F).

### Alpha diversity analysis

**Fig 4:**
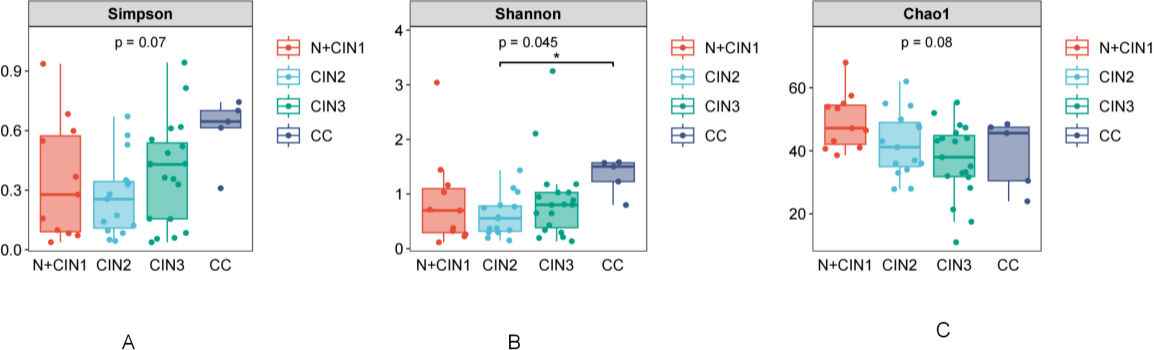
Alpha Diversity Indices Across Cervical Lesion Groups (**A**) Simpson Index (evaluating community dominance and evenness; lower values indicate reduced diversity). (**B**) Shannon Index (integrating species richness and evenness; higher values denote greater diversity). (**C**) Chao1 Index (estimating total species richness; higher values correspond to greater richness). Boxplots display median values, interquartile ranges, and outliers.

The Simpson index (Fig. 4A) revealed stratified microbial diversity across lesion groups, with the CC group exhibiting the highest mean value, followed by CIN3. The N + CIN1, and CIN2 groups had statistically similar diversity scores, although no significant overall differences were observed across all groups (P=0.07, Kruskal-Wallis test).

In contrast, the Shannon index (Fig. 4B) showed significantly greater microbial diversity in the CC group compared to the CIN2 group (P=0.045, Dunn post-hoc test), with CIN3 and CIN2 displaying a sequential decrease in diversity.

Notably, the Chao1 index (Fig. 4C) indicated no statistically significant variations in species richness across groups (overall P=0.08). Although the N + CIN1 group exhibited the highest numerical values, post-hoc tests did not reveal significant pairwise differences. A trend of declining richness was observed from the Normal group to CIN3, while the CC group showed slightly elevated values compared to CIN3 but remained lower than the N + CIN1 group.

### Beta diversity analysis

**Fig 5:**
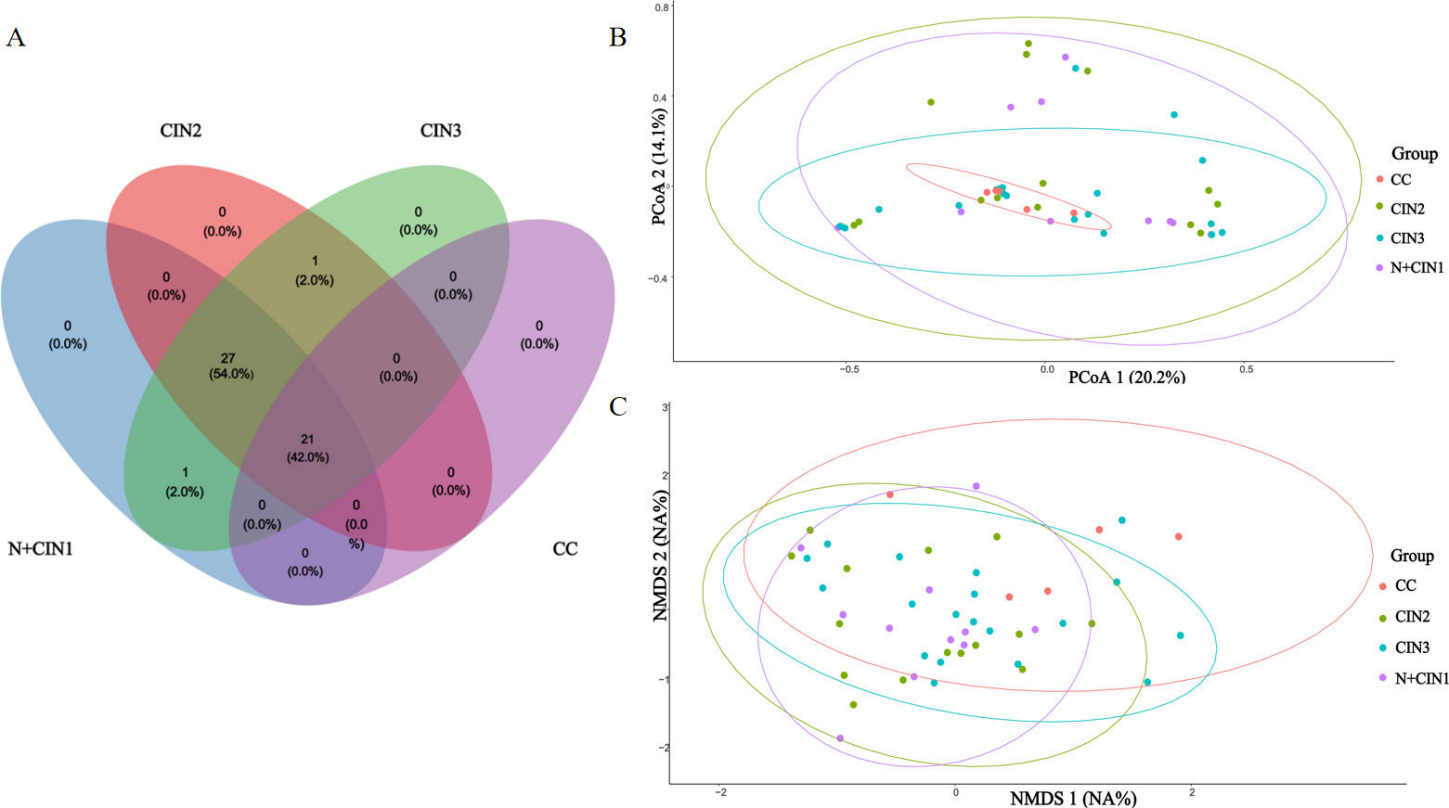
Cervical Microbiota Composition and Beta Diversity Across Cervical Lesion Groups (**A**) Venn diagram illustrating shared and unique Operational Taxonomic Units (OTUs) among the study groups (N + CIN1, CIN2, CIN3, and CC), with a focus on overlaps in microbial taxa. (**B**) PCoA based on weighted UniFrac distances—incorporating both phylogenetic similarity and abundance—visualizing differences in microbial community structure across groups. (**C**) NMDS ordination depicting beta diversity patterns derived from overall microbial composition dissimilarity (Bray-Curtis distance). Points positioned closer together indicate more similar microbial communities.

Fig. 5 depicts community compositional shifts in the cervical microbiota across lesion stages. Venn diagram analysis (Fig. 5A) showed that 54% of species-level OTUs were shared among the N + CIN1, CIN2, and CIN3 groups, whereas only 42% of OTUs were common across all four groups (N + CIN1, CIN2, CIN3, and CC).

Beta diversity analyzes using weighted UniFrac-based PCoA (Fig. 5B) and NMDS (Fig. 5C) revealed no statistically significant differences among the four groups (P>0.05 for both analyzes), indicating minimal compositional dissimilarity in microbial communities despite increasing lesion severity.

### Comparison of cervical microbial community differences

**Fig 6:**
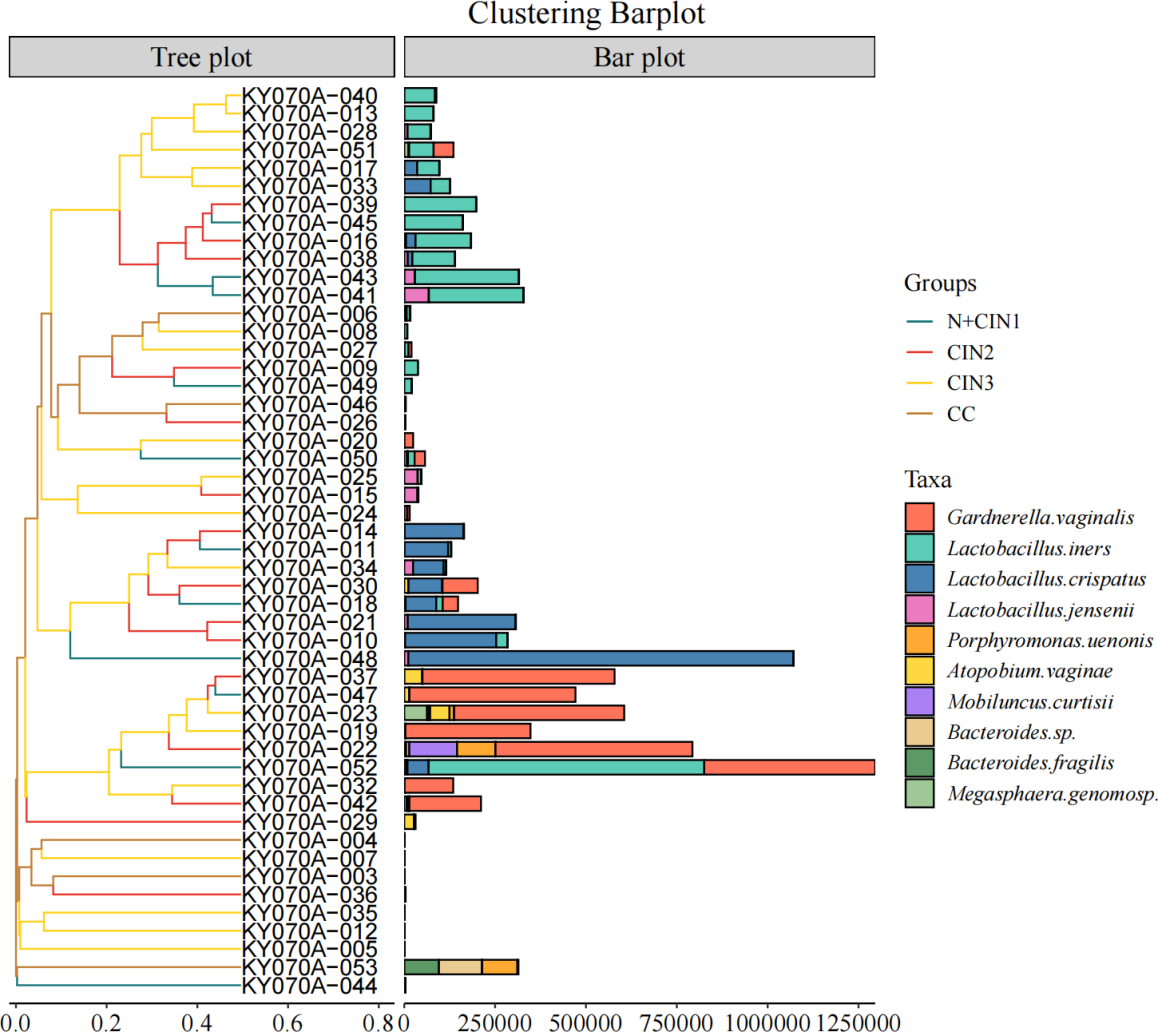
Hierarchical clustering of cervical microbial communities across lesion stages. In the left-hand tree plot, the y-axis displays individual sample identifiers, which are clustered based on microbial community similarity. In the right-hand bar plot, the y-axis lists microbial taxa, while the x-axis features dual abundance scales: Upper scale: Relative abundance (normalized proportion, ranging from 0.0 to 0.8). Lower scale: Absolute abundance (sequence counts, ranging from 0 to 1,250,000).

**Fig 7:**
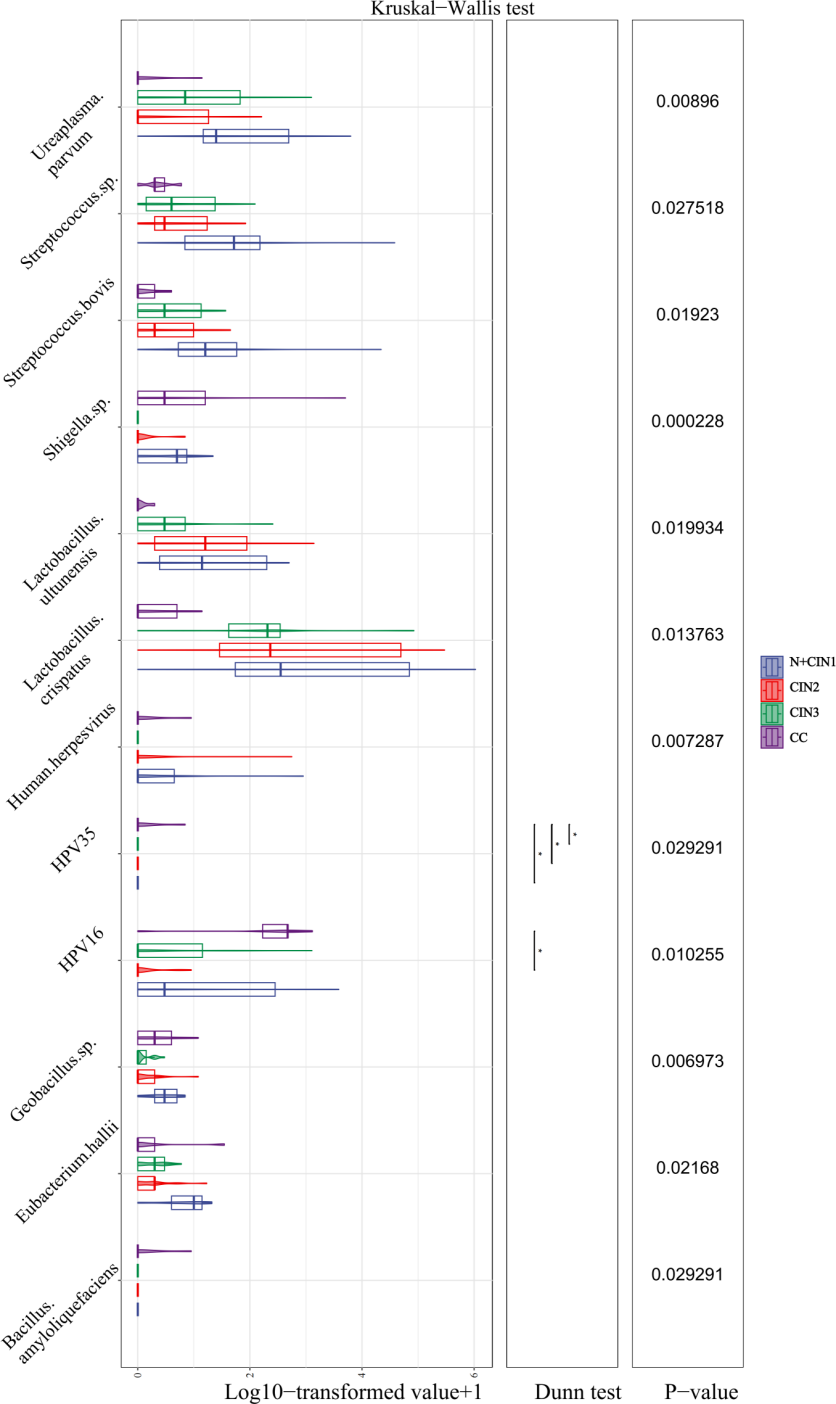
Microbial abundance across cervical lesion stages. Boxplots display log10-transformed (values + 1) microbial abundance in four groups: normal/CIN1 (N + CIN1, blue), CIN2 (red), CIN3 (green), and CC (purple). The Kruskal-Wallis H test was used for overall comparisons, with significant Dunn’s post-hoc pairwise tests indicated by connecting lines and corresponding P-values (*P < 0.001; *P* < 0.01; *P < 0.05).

Fig. 6 shows hierarchical cluster analysis of species-level microbial compositions, revealing close compositional similarities between the N + CIN1 and CIN2 groups. *Lactobacillus iners* and *Lactobacillus crispatus* were identified as key discriminative biomarkers driving this similarity. Subsequent Kruskal-Wallis H tests with Dunn’s post-hoc comparisons (Fig. 7) identified *L. ultunensis* and *L. crispatus* as potential biomarkers for CIN, demonstrating significantly reduced abundance in the CC group (P<0.01). Notably, HPV16 and HPV35 were strongly correlated with CC, highlighting their potential synergistic role with microbial dysregulation in cervical carcinogenesis.

### Identification of differential microbial biomarkers via LEfSe analysis

**Fig 8:**
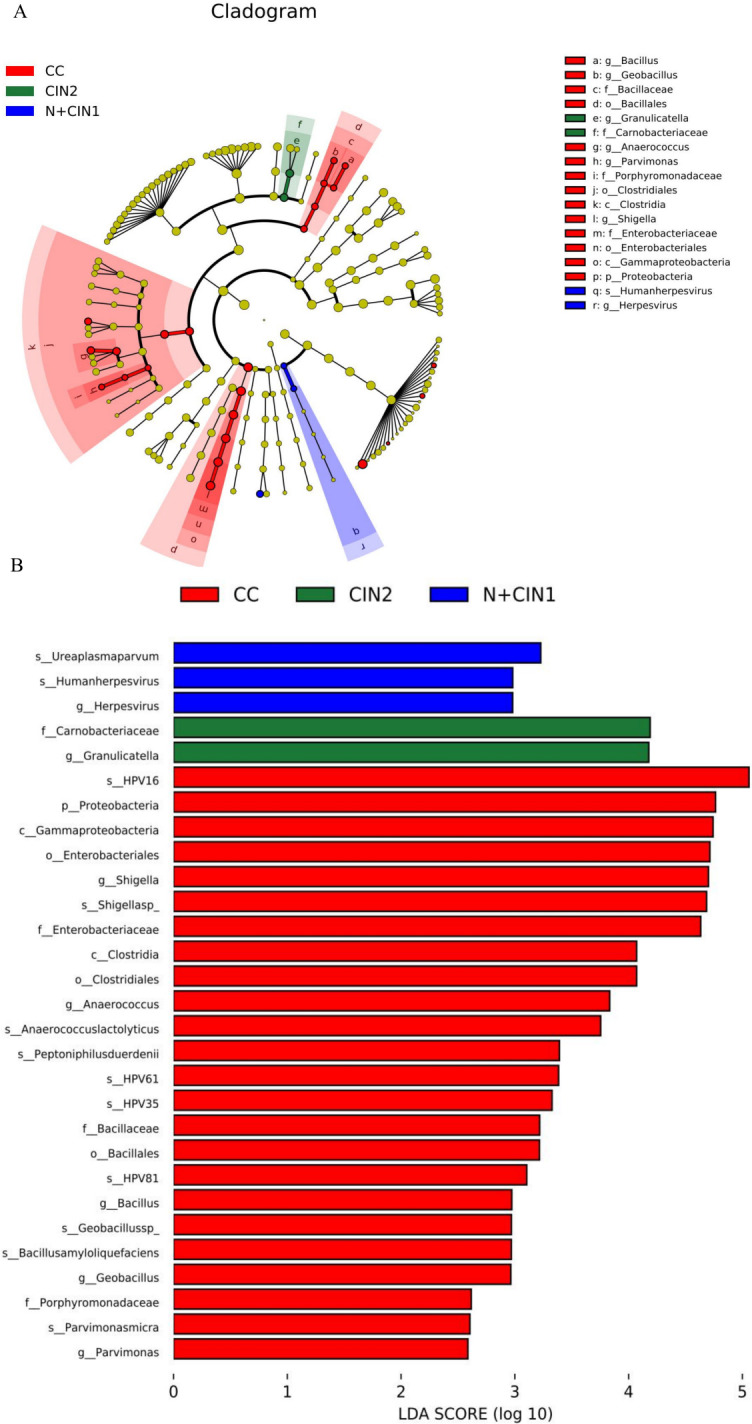
LEfSe analysis identifies differential microbial biomarkers across cervical lesion stages. (**A**) LEfSe cladogram depicting the cervical microbiota across lesion groups. Microbial taxa (from phylum to genus) are represented by concentric circles radiating from the center, with branch lengths proportional to taxonomic hierarchy . (**B**) Histogram of LDA scores for microbial taxa identified as significant biomarkers (threshold: *P* < 0.05, LDA score >2). Taxa are ranked by descending LDA scores, reflecting their discriminatory power across lesion stages.

LEfSe analysis was employed to identify microbial taxa with statistically significant abundance variations across the four groups (LDA score >2.0, P<0.05). As shown in Fig. 8, species-level analysis revealed two key discriminative biomarkers in the N + CIN1 group: *Ureaplasma parvum* and Human herpesvirus. In contrast, the CIN2 group was characterized by enrichment of the family Carnobacteriaceae and the genus *Granulicatella*. Notably, the CC group exhibited the most discriminative microbial features, including species such as *Parvimonas micra*, *Bacillus amyloliquefaciens*, *Geobacillus sp.*, HPV16, HPV81, HPV35, HPV61, *Peptoniphilus durdenii*, *Anaerococcus lactolyticus*, and *Shigella sp.* at the species level.

### Random forest-derived ranking of top 20 microbial biomarkers for cervical lesion stages

**Fig 9:**
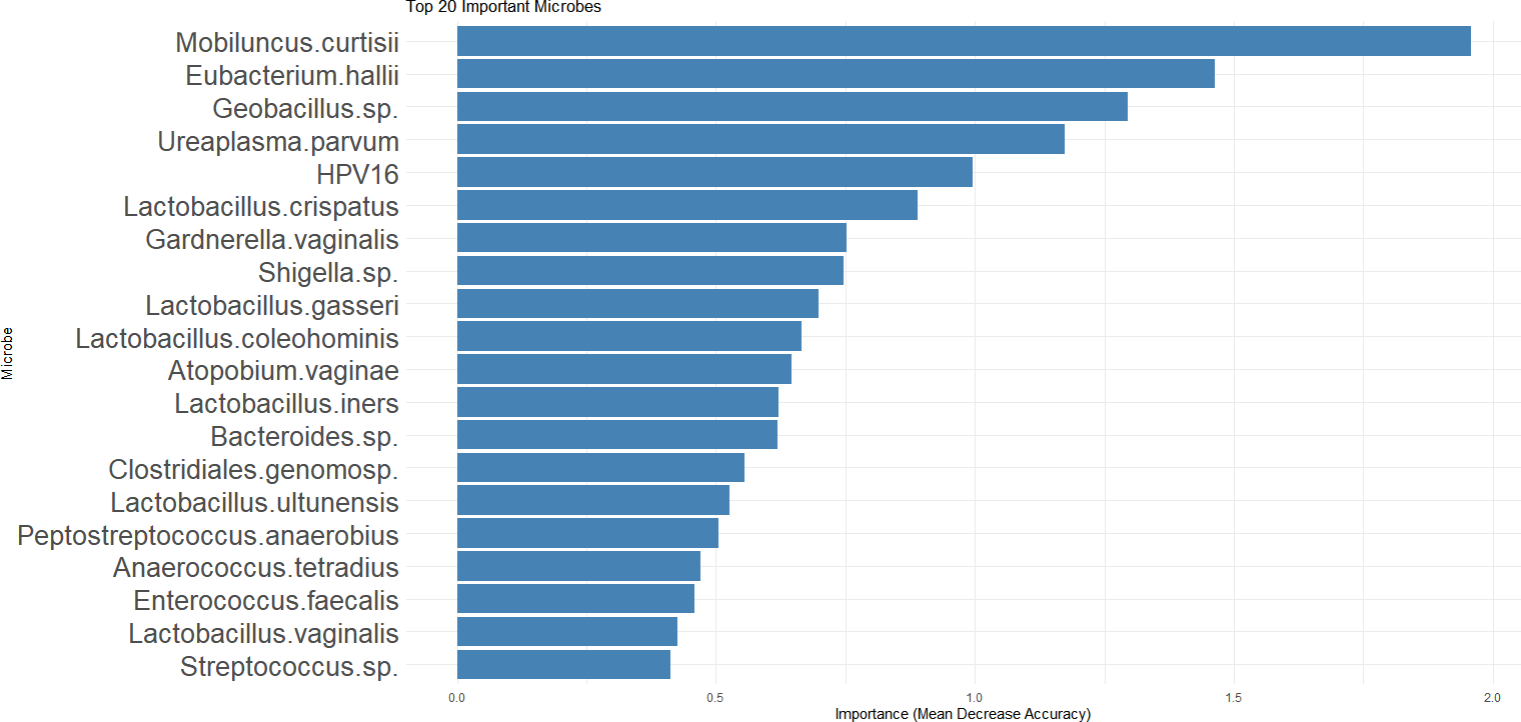
Top 20 Important Microbes in Cervical Lesion Analysis. Y-axis: Lists the top 20 microbial taxa identified as important biomarkers via Random Forest modeling. Taxa are ordered by their importance scores (descending). X-axis: Importance score (Mean Decrease Accuracy), quantifying a microbe’s discriminatory power to distinguish cervical lesion stages (N + CIN1, CIN2, CIN3, CC). Higher values indicate stronger contribution to classifying lesion stages.

LEfSe-based identification of human herpesvirus in the N + CIN1 group was driven by its abundance in a small subset of participants. Given the constraints of sample size, the statistical significance of this finding warrants cautious interpretation. To address this limitation, we performed supplementary random forest analyzes to validate these results. Random Forest modeling identified the top 20 microbial taxa as important biomarkers for distinguishing cervical lesion stages . As shown in Fig. 9, *Mobiluncus curtisii* exhibited the highest importance score (Mean Decrease Accuracy), indicating its prominent role in differentiating among lesion stages. Among the ranked taxa, *Eubacterium hallii* and *Geobacillus sp.* also showed relatively high importance. Notably, HPV16 was included in the list, highlighting the involvement of viral-microbial interactions in cervical lesion progression. *Lactobacillus* species, such as *L. crispatus*, *Lactobacillus gasseri*, and others, were identified as important biomarkers, reflecting their complex roles in maintaining microbial balance or contributing to dysbiosis during the development of cervical lesions. In general, these top 20 microbial taxa, with their varying importance scores, provide insights into the microbial community associated with different cervical lesion stages and lay a foundation for further exploring the mechanisms of cervical lesion development and potential microbial-based diagnostic or therapeutic targets.

## DISCUSSION

Our study comprehensively examined cervical microbiota across the cervical disease spectrum, from benign conditions to invasive cervical carcinoma, using species-level LC-WGS and a custom-developed bioinformatics pipeline named UCAD. Our findings corroborate established associations between *Lactobacillus* depletion, increased microbial diversity, and CIN progression to CC (13, 14). Specifically, we observed a progressive decline in *Lactobacillus* dominance (e.g., *L. crispatus*) alongside the enrichment of pathobionts such as *Gardnerella* during the CIN1-to-CC transition. Notably, the microbial divergence between CIN and CC cohorts suggests potential microbial contributions to invasive progression, thereby warranting mechanistic exploration of microbiota-driven oncogenic pathways.

The functional roles of distinct *Lactobacillus* species within the CM remain controversial, with emerging evidence highlighting species-specific effects on cervical health. While *L. crispatus* and *L. gasseri* are commonly associated with a protective vaginal environment through acidification and anti-inflammatory mechanisms, *L. iners* has been linked to increased inflammation and heightened susceptibility to bacterial vaginosis (4, 15). Notably, Arokiyaraj et al. reported contrasting roles: *L. johnsonii* correlated with HPV persistence, whereas *L. crispatus* dominance was observed in individuals achieving HPV clearance (15), suggesting that species-level taxonomic resolution is essential for understanding microbial-HPV interactions. Consistent with these findings, our data revealed substantial relative abundances of *Lactobacillus* spp. in the CM, further emphasizing the need to delineate species-specific contributions rather than treating the genus as a homogeneous group in future mechanistic and therapeutic investigations.

Multiple studies have demonstrated that HPV infection is associated with an increased abundance of specific bacterial genera, including *Bifidobacterium*, *Bacillus*, *Megasphaera*, *Prevotella*, and *Gardnerella*, which are more prevalent in HPV-positive populations compared to healthy controls (16). This relationship suggests a bidirectional feedback loop: HPV infection exacerbates cervical microbiota dysbiosis, while the altered microbial environment further promotes viral persistence and progression, ultimately driving the development of advanced cervical lesions. However, conflicting evidence exists, as a report (17) proposes that HPV alone may be insufficient to induce substantial shifts in cervicovaginal microbiota composition. In our analysis, HPV16 and HPV35 emerged as significant biomarkers in the CC group, which exhibited the highest alpha diversity indices (Simpson and Shannon) among all cohorts. Notably, a statistically significant difference in Shannon index values was observed between the CC and CIN2 groups (P=0.045), highlighting both the established association between hr-HPV infection and cervical carcinogenesis and a potential link between HPV-driven microbial community restructuring and elevated cervical microbial diversity during disease progression.

A key advancement lies in overcoming 16S rRNA sequencing’s taxonomic limitations via LC-WGS-based species-level resolution profiling, enabling detection of bacteria, fungi, and viruses. Rigorous exclusion of confounders (ethnicity, sexual activity, hygiene practices) and the use of age-matched cohorts strengthen internal validity (18). A small sample size represents a key limitation, necessitating multi-center validation. While Simpson and Chao1 indices showed no significant differences in microbial richness or diversity between groups, the Shannon index revealed significant disparities between CIN2 and CC groups, potentially reflecting rare species uniquely captured by this index and warranting confirmation via expanded sampling. LEfSe identified Human herpesvirus as a biomarker in the N + CIN1 group due to its abundance in the few participants, yet Random Forest excluded it from top discriminative microbes, indicating limited robustness. In contrast, Ureaplasma parvum and HPV16 remained significant across methods, supporting biological relevance. The cross-sectional design also precluded distinguishing persistent versus transient HPV infections, further limiting causal inferences and emphasizing the need for longitudinal studies. Future cohort tracking and integrated approaches will address these limitations to validate findings and reduce small-sample size biases.

## Data Availability

The raw count data are provided in Table S1. Raw low-coverage whole-genome sequencing (LC-WGS) data have been deposited in the Genome Sequence Archive (Genomics, Proteomics & Bioinformatics 2021) in National Genomics Data Center (Nucleic Acids Res 2022), China National Center for Bioinformation / Beijing Institute of Genomics, Chinese Academy of Sciences (GSA: CRA028360) that are publicly accessible at https://ngdc.cncb.ac.cn/gsa (19, 20).

## SUPPLEMENTAL MATERIAL

Table S1 spectrum.03206-24-s0001.xlsx Microbial abundance.

ASM does not own the copyrights to Supplemental Material that may be linked to, or accessed through, an article. The authors have granted ASM a non-exclusive, world-wide license to publish the Supplemental Material files. Please contact the corresponding author directly for reuse.
